# Prospective study on Maresin-1 and cytokine levels in medication-naïve adolescents with first-episode major depressive disorder

**DOI:** 10.3389/fpsyt.2023.1132791

**Published:** 2023-03-15

**Authors:** Tian Qiu, Xiao Li, Wanjun Chen, Jinglan He, Lei Shi, Chenxi Zhou, Anhai Zheng, Zhongli Lei, Chenglu Tang, Qingchan Yu, Lian Du, Jiamei Guo

**Affiliations:** Department of Psychiatry, The First Affiliated Hospital of Chongqing Medical University, Chongqing, China

**Keywords:** major depressive disorder, adolescent, Maresin-1, cytokines, medication-naïve

## Abstract

**Background:**

Inflammation and immune activation may play a role in the pathological mechanism of Major Depressive Disorder (MDD). Evidence from cross-sectional and longitudinal studies of adolescents and adults has shown that MDD is associated with increased plasma pro-inflammatory cytokines (e.g., IL-1β, IL-6). It has been reported that Specialized Pro-resolving Mediators (SPMs) mediate inflammation resolution, and Maresin-1 can activate the process of inflammation and promote inflammation resolution by promoting macrophage phagocytosis. However, no clinical studies have been conducted to evaluate the relationship between the levels of Maresin-1 and cytokine and the severity of MDD symptomatology in adolescents.

**Methods:**

40 untreated adolescent patients with primary and moderate to severe MDD and 30 healthy participants as the healthy control (HC) group aged between 13 and 18 years old were enrolled. They received clinical and Hamilton Depression Rating Scale (HDRS-17) evaluation and then, blood samples were collected. Patients in the MDD group were re-evaluated for HDRS-17, and blood samples were taken after a six to eight-week fluoxetine treatment.

**Results:**

The adolescent patients with MDD had lower serum levels of Maresin-1 and higher serum levels of interleukin 6 (IL-6) compared with the HC group. Fluoxetine treatment alleviated depressive symptoms in MDD adolescent patients, which was reflected by higher serum levels of Maresin-1 and IL-4 and lower HDRS-17 scores, serum levels of IL-6, and IL-1β. Moreover, the serum level of Maresin-1 was negatively correlated with the depression severity scores on the HDRS-17.

**Conclusion:**

Adolescent patients with primary MDD had lower levels of Maresin-1 and higher levels of IL-6 compared with the HC group, implying that the peripheral level of pro-inflammatory cytokines may be elevated in MDD, resulting in the insufficiency of inflammation resolution. The Maresin-1 and IL-4 levels increased after anti-depressant treatment, whereas IL-6 and IL-1β levels decreased significantly. Moreover, Maresin-1 level negatively correlated with depression severity, suggesting that reduced levels of Maresin-1 promoted the progression of MDD.

## 1. Introduction

Major Depressive Disorder (MDD) is a severe mental disorder characterized primarily by significant and persistent depression that affects about 322 million people globally (1). Depression is more prevalent among adolescents (2), with an 8% annual prevalence (3) and a lifetime prevalence of 11% in this age group (4). Adolescents with MDD are at a high risk of self-injury and suicide. Hence, adolescent MDD has been the leading cause of adolescents’ severe physical and mental health, with serious negative impacts on families, communities, and society, becoming the cause of the burden of disease in this age group (5). Unfortunately, the adolescents’ response rate to psychotherapy and antidepressant medication is not optimistic. For instance, adolescent MDD patients had a far lower response to antidepressant medication treatment than adult with MDD. Among 10 common antidepressant treatments in clinical practice, a large mesh meta-analysis showed that only Fluoxetine was beneficial to adolescent MDD, whereas the other antidepressants were no more effective than the placebo (6, 7). A better understanding of the underlying pathophysiological mechanisms associated with adolescent MDD and depressive symptoms is critical for improving the efficacy of existing treatments, developing new therapies, and selecting patients who will benefit the most from the treatments targeting these biological pathways.

Although several risk factors and etiological models of MDD have been proposed, recent studies have focused on the role of immune dysregulation, one of the most important mechanisms in MDD pathophysiology. Pro-inflammatory cytokines, in particular, are tiny signal proteins that coordinate the innate inflammation response (8, 9). It has been demonstrated that pro-inflammatory cytokines can regulate the activation of the hypothalamus-pituitary axis and enter the central nervous system to affect MDD-related neural circuits and neurotransmitter functioning (10). Previous studies found that adult patients with MDD showed relatively high peripheral levels of cytokines, such as Interleukin (IL)-6, C-Reactive Protein (CRP), and Tumor Necrosis Factor α (TNF-α) (11–13). The immune system undergoes considerable growth during adolescence, including the shrinkage of lymphoid tissues and changes in sex hormones (14). Moreover, inflammation may play a role in the pathophysiology of adolescent MDD. Unfortunately, current research on the relationship between inflammation and adolescent MDD is still inadequate, with inconsistent results. A latest study found that the levels of IL-2, interferon γ (IFN-γ), TNF-α, and IL-10 in MDD adolescent patients were lower at baseline but higher after the treatment compared with the Healthy Control (HC) group (15). However, another study reported higher levels of IL-4, IL-10, and TNF-α in adolescent patients with MDD compared with the HC group. Other studies found no significant differences in levels of inflammatory factors (IL-1β, IL-4, IL-6, CRP, and TNF-α) between the MDD and HC groups (16–18). A recent meta-analysis reported that after receiving a routine anti-depressant treatment, adult MDD patients had significantly lower levels of IL-6, TNF-α, and IL-10 (19). The SSRI treatment reduced the level of at least one cytokine (20–22). Nevertheless, these results contradict each cytokine’s pro-inflammatory or anti-inflammatory properties. In conclusion, research focusing on a single inflammatory component in adolescent patients with MDD considering has certain limitations (16, 23).

Inflammation is the fundamental response to protect the host from harmful external antigens and microorganisms. Excessive inflammation responses, on the other hand, cause tissue damage and impair organ function. Therefore, the anti-inflammatory effect and the mechanism of inflammation resolution should be investigated. The inflammation resolution is currently regarded as a dynamic, highly programmed response that includes the end of the inflammation response and the recovery of tissue homeostasis (24, 25). Inflammation resolution is a biological process strictly regulated by Specialized Pro-resolving Mediators (SPMs) to terminate inflammation response and promote spontaneous repair. The SPM family is a kind of endogenous active substance derived from Omega-6 (arachidonic acid, AA) and Omega-3 (eicosapentaenoic acid, EPA; docosahexaenoic acid, DHA), which have a fatty acid with similar biological activity and promote the inflammation resolution (26). Maresin-1 is one of the metabolites derived from docosahexaenoic acid (DHA), which has been shown to have various intensively anti-inflammatory effects in inflammatory diseases (27). Maresin-1 was discovered in human macrophages and is an active lipid mediator during inflammation resolution. Recent studies have demonstrated that reducing Maresin-1 causes inflammation resolution failure, leading to chronic inflammatory diseases, including atherosclerosis (26), dementia (28, 29), and diabetes nephropathy (30). Patients with these chronic inflammatory diseases typically develop depressive symptoms or have an increased risk of MDD comorbidity. However, whether this indicates an inflammation resolution disorder in patients with MDD has not been determine. A recent preclinical study found that intracerebroventricular injection of SPMs can significantly reduce the depressive behavior of mice induced by lipopolysaccharide (LPS) (31, 32). A series of studies have suggested a potential association between immune response disorder and abnormal inflammation resolution in MDD patients.

This study investigated the alterations in the cytokine profiles, Maresin-1, and the symptomatology in medication-naïve adolescents with first-episode MDD. We hypothesized that these adolescents’ Maresin-1 and inflammatory markers differed significantly from those of healthy controls and that these differences changed after antidepressant treatment. We also looked at the correlation between the severity of depression and levels of Maresin-1 and cytokines. We investigated the relationship between depression in adolescence and the immune response and compared our findings with existing findings.

## 2. Materials and methods

### 2.1. Subjects

From January 2021 to November 2021, the adolescent psychological department of the First Affiliated Hospital of Chongqing Medical University assessed 114 patients. Of these, 40 patients who met the inclusion criteria were enrolled and finished this longitudinal intervention follow-up study. The inclusion criteria involved primary and untreated cases conforming to the diagnostic standards of DSM-V moderate monophasic depression. All patients agreed to participate in the study and signed informed consent form. The study also recruited 30 healthy individuals aged between 13 and 18 years as the HC group. The exclusion criteria were a history of manic or hypomanic episodes; a history of cerebral brain diseases or severe brain trauma; patients with heart, liver, and kidney diseases, diabetes, and other strenuous physical diseases; a history of substance dependence or abuse (e.g., tobacco, alcohol, cocaine, drugs); those who refused to participate in the research; and the individuals with intellectual disabilities.

### 2.2. Clinical procedures

The SCID-I/P clinical stereotype questionnaire was used to diagnose all patients. At least two independent, experienced psychiatrists diagnosed patients using DSM-V as the diagnostic standard (33). Patients with moderate to severe MDD received Fluoxetine at the initial dose of 20 mg/day, adjusted to 20–40 mg/day based on the condition evaluation during the follow-up. The general data collection, blood sample collection and HDRS-17 scale (Hamilton depression rating scale (HDRS)) (34) evaluation of the enrolled patients were conducted at baseline, and clinical symptom scale evaluation and blood sample collection were repeated after 6–8 weeks of treatment. The clinical symptoms were evaluated, and blood samples were taken only at the enrolment in the HC group. This study complies with medical ethical standards and has been approved by the Ethics Committee of the First Affiliated Hospital of Chongqing Medical University. Before the study, the personnel conducting the clinical scale evaluation were trained on the consistency of the scale and subsequently passed a consistency assessment test.

### 2.3. Sample collection, detection of Maresin-1, and routine biochemical measurement

After 8–10 h of fasting, 5 mL of fasting venous blood was collected from each participant in the patient and HC groups using anticoagulant-free vacuum tubes. The blood samples were centrifuged for 5 min at 3000 rpm to separate serum after standing for 1–2 h at room temperature, then stored at −80°C. Enzyme-Linked- Immunosorbent Serologic Assay (ELISA) was performed to detect the serum levels of Maresin-1, IL-1β, IL-6, and IL-4 according to the operating procedure of the manufacturer. Serum levels of Maresin-1, IL-1β, IL-6, and IL-4 were measured using Human Maresin-1 ELISA Kit (LCSKIT, ED-15413), Human IL-1βELISA kit (LCSKIT, ED-10351), Human IL-6 ELISA kit (LCSKIT, ED-10377), and Human IL-4 ELISA kit (LCSKIT, ED-10375) according to the manufacturer’s recommendations.

### 2.4. Statistical analysis

We performed statistical analysis using SPSS version 22. First, we calculated the means and standard deviations of the demographic and clinical data. The Intra-Assay Coefficient of Variability (CV) was calculated to represent the repeatability of ELISA test results. The average of the individual CVs is reported as the intra-assay CV. To compare the levels of Maresin-1 and each cytokine between the MDD and Control groups, we performed a normality test and then analyzed the normal distribution data using the student test or the Mann–Whitney test, as appropriate. We also performed paired t-tests to measure changes in Maresin-1 and cytokine levels in the MDD group before and after treatment. Finally, we analyzed Pearson correlations and linear regression analyses in Maresin-1, each cytokine level, and the severity of depression. Statistical significance was set at *p* < 0.05.

## 3. Results

### 3.1. Participants and demographic data

The 40 adolescent patients with primary and untreated MDD comprised 13 males and 27 females, with an average age of 15.68 ± 1.42 years and an average BMI of 20.07 ± 2.55. The HC group included 15 males and 15 females, with an average age of 16.13 ± 1.31 and an average BMI of 20.28 ± 4.16. Before treatment, the HDRS-17 scores of the adolescent MDD patients and the HC group were 25.05 ± 5.91 and 3.07 ± 2.18, respectively. The HDRS-17 depression scores were significantly different, whereas age, gender, and BMI did not vary much between the two groups (Table 1). Hence, the two groups were comparable.

**Table 1:** Demographic and clinical data.

	MDD adolescents (n = 40)	Healthy control (n = 30)	t/F	p-Value
Age (years, M ± SD)	15.68 ± 1.42	16.13 ± 1.31	1.38	0.172
Gender (male/female)	13/27	15/15	1.452	0.228
BMI (kg/m2, M ± SD)	20.07 ± 2.55	20.28 ± 4.16	0.251	0.802
HDRS (M ± SD)	25.05 ± 5.91	3.07 ± 2.18	52.13	<0.001*

For continuous variables, statistical analysis was performed by Student’s T-test. For categorical variables, the p-value was calculated using the chi-square test.

*Statistically significant: *p* < 0.05.

### 3.2. Serum levels of Maresin-1 and cytokines of adolescent patients with MDD and The HC group

**Figure 1:**
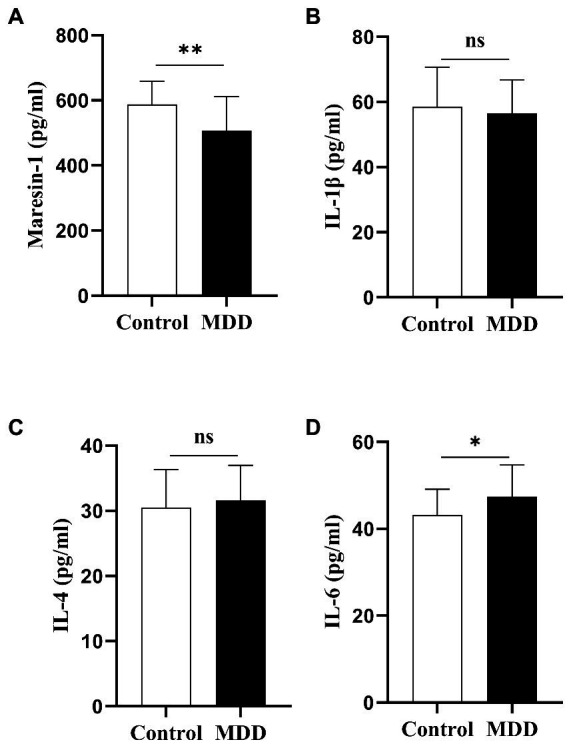
The cytokine serum level and Maresin-1 serum level comparison between adolescents with MDD healthy Controls and adolescents with MDD at baseline. *p < 0 05; **p < 0 01. *p*-Value was calculated using the *t*-test was performed for statistical analysis.

The serum levels of Maresin-1 and cytokines of adolescent patients with MDD and the HC group were investigated. All the average of the individual CVs for maresin-1 and cytokines in adolescent patients with MDD and HCs was less than 10%. Compared with the HC group, the level of Maresin-1 in adolescent MDD patients was significantly lower (*p* < 0.001, Table 2, Figure 1A)and the level of IL-6 was significantly higher (*p* < 0.05, Table 2, Figure 1D). However, there were no significant differences in the levels of IL-1β and IL-4 between the two groups (*p* > 0.05, Table 2, Figure 1B,C), implying that serum levels of Maresin-1 and inflammatory cytokines IL-6 might be associated with adolescent MDD patients.

**Table 2:** Serum levels of Maresin-1 and cytokines of adolescent patients with MDD and the HC group.

	MDD adolescents (*n* = 40)		Healthy control (*n* = 30)		*t*/*Z*	*p*-Value
	M ± SD	intra-assay %CV	M ± SD	intra-assay %CV		
Maresin-1 (pg/mL)	506.95 ± 104.85	3.23	587.74 ± 71.39	2.63	−3.063	0.002*
IL-1β (pg/mL)	56.47 ± 10.29	3.25	58.50 ± 12.23	2.02	−1.021	0.307
IL-4 (pg/mL)	31.57 ± 5.40	1.38	30.48 ± 5.84	3.47	−0.878	0.38
IL-6 (pg/mL)	47.44 ± 7.29	1.92	43.15 ± 5.99	2.39	2.622	0.011*

For continuous variables, the p-Value was calculated using Student’s T-test for normally distributed or the Mann–Whitney nonparametric test for nonnormally distributed.*Statistically significant: *p* < 0.05.

### 3.3. Levels of Maresin-1 and cytokine in adolescent patients with MDD after treatment

**Figure 2:**
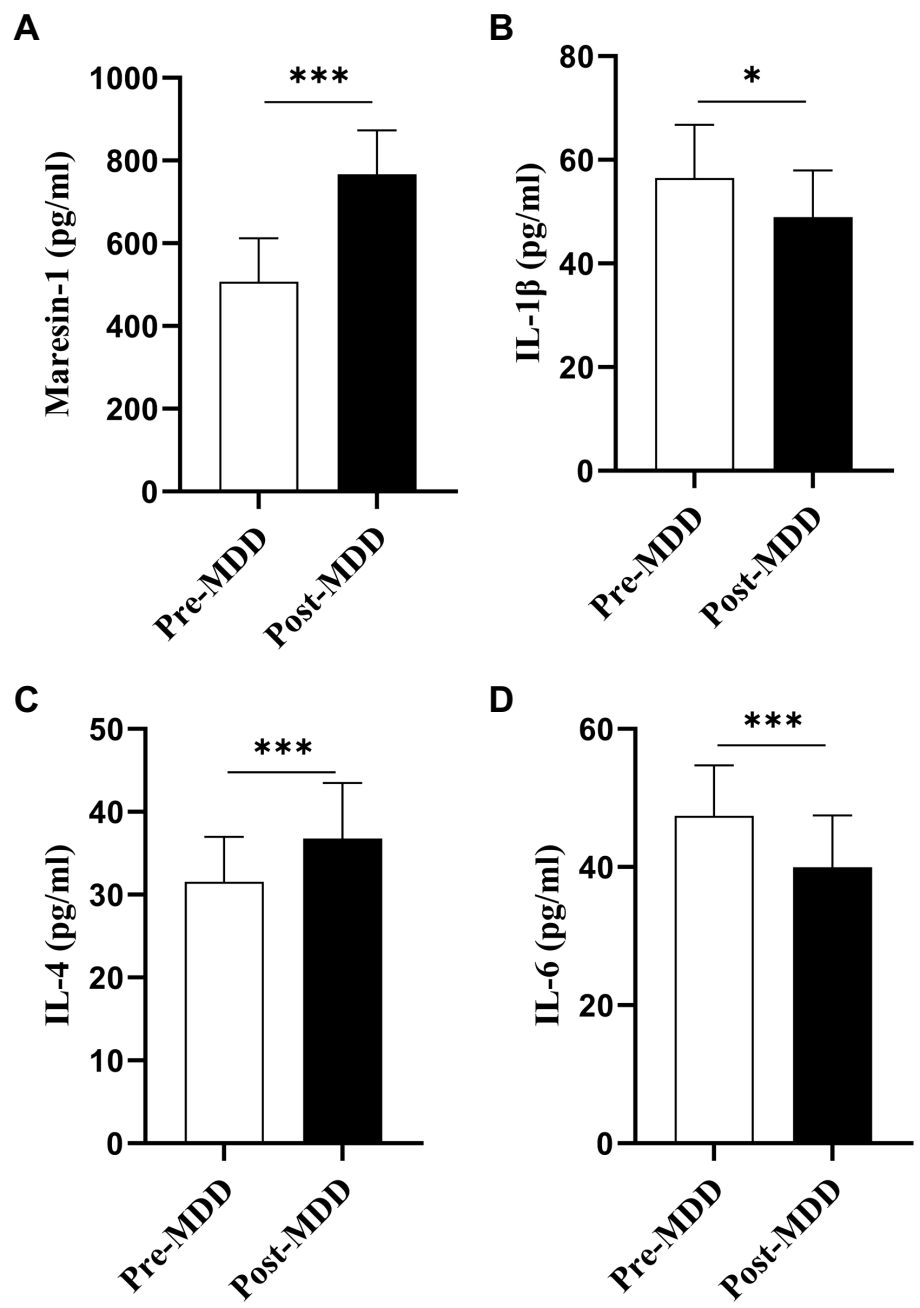
Pre-MDD: before the fluoxetine treatment. Post-MDD: after the fluoxetine treatment. Values are presented as mean ± standard deviation for continuous variables, *p*-Value was calculated using the *t*-test *Statistically significant: *p* < 0.05.

A first-line clinical antidepressant, fluoxetine 20-40 mg/day, was administered to 40 MDD adolescent patients. All the average of the individual CVs for maresin-1 and cytokines in adolescent patients was less than 10%. The Maresin-1 serum levels and several key cytokines were dynamically evaluated before and after 6-8-week anti-depressant treatment. First, after fluoxetine treatment, the serum level of Maresin-1 was significantly higher than that before treatment (*p* < 0.001, Table 3, Figure 2A). For the three examined cytokines, compared with levels before treatment, IL-1β (*p* < 0.05, Table 3, Figure 2B) and IL-6 (*p* < 0.001, Table 3, Figure 2D) and the score of HDRS-17 were significantly decreased (*p* < 0.001, Table 3), whereas IL-4 increased dramatically after treatment (*p* < 0.001, Table 3, Figure 2C). Hence, fluoxetine treatment reversed depressive symptoms, decreased Maresin-1 serum levels, increased IL-6 in MDD adolescent patients, and significantly down-regulated IL-1β and up-regulated IL-4, as shown in Table 3.

**Table 3:** Levels of Maresin-1 and cytokine in adolescent patients with MDD after treatment.

	Pre-MDD (n = 40)		Post-MDD (n = 40)		*t*/*Z*	*p*-Value
	M ± SD	intra-assay %CV	M ± SD	intra-assay %CV		
HDRS	25.05 ± 5.91	-	11.15 ± 2.87	-	12.83	<0.001*
Maresin-1 (pg/mL)	506.95 ± 104.85	3.23	767.12 ± 106.10	3.38	−5.512	<0.001*
IL-1β (pg/mL)	56.47 ± 10.29	3.25	48.97 ± 9.019	2.88	−2.353	0.019*
IL-4 (pg/mL)	31.57 ± 5.40	1.38	36.78 ± 6.69	2.12	−4.008	<0.001*
IL-6 (pg/mL)	47.44 ± 7.29	1.92	39.96 ± 7.50	1.15	−4.069	<0.001*

### 3.4. Correlation among depressive symptom severity and levels of Maresin-1 and cytokines in adolescent patients with MDD

The relationship between depressive symptom severity and the levels of Maresin-1 and three cytokines in adolescent MDD patients was evaluated. The Pearson correlation analysis results are shown in Table 4. The results demonstrated that Maresin-1, IL-1β, IL-4, and IL-6 were significantly correlated with the HDRS-17 score. Multiple linear regression analysis revealed that the HDRS-17 Score was independently negatively correlated with the serum level of Maresin-1 (standardized β = −0.618, *p* < 0.000). The HDRS-17 Score was independently positively correlated with the serum level of IL-6 (standardized β = 0.162, *p* < 0.05) and IL-1β (standardized β = 0.173, *p* < 0.05) (Table 5).

**Table 4:** The Pearson correlation analysis among depressive symptom severity and levels of Maresin-1 and cytokines.

	HDRS	Maresin-1	IL-1β	IL-4	IL-6
HDRS	1	-	-	-	-
Maresin-1	−0.725***	1	-	-	-
IL-1β	0.366**	−0.329**	1	-	-
IL-4	−0.421***	0.479***	−0.289**	1	-
IL-6	0.397 ***	−0.444***	0.018	−0.125	1

**Statistically significant: *p* < 0.01; *** Statistically significant: *p* < 0.001.

**Table 5:** Multiple linear regression analysis among depressive symptom severity and levels of Maresin-1 and cytokines.

Predictors	*B*	SE	*Β*	*T*	*p* Value
Maresin-1	−0.031	0.005	−0.618	−6.765	0.000*
IL-1β	0.140	0.061	0.173	2.316	0.023*
IL-4	−0.122	0.101	−0.096	−1.206	0.232
IL-6	0.164	0.079	0.162	2.064	0.043*

*Statistically significant: *p* < 0.05.

## 4. Discussion

The inflammatory factor IL-6 was higher in the peripheral blood of adolescent MDD patients compared with the HC group, which was consistent with previous studies. However, we first found a lower level of Maresin-1, indicating that adolescent MDD patients were exposed to pro-inflammatory effects and insufficient inflammation resolution. The HDRS-17 Score in adolescent MDD patients decreased significantly after treatment with Fluoxetine, suggesting reduced depression severity. Similarly, after treatment, IL-1β and IL-6 were significantly lower, whereas the level of Maresin-1 was substantially higher. We first reported a significant difference in the level of Maresin-1 between adolescent MDD patients and the HC group. The serum level of Maresin-1 was negatively correlated with depressive symptoms, suggesting that Maresin-1 is a potential novel marker for predicting disease progression and treatment effect.

Adolescence is a critical physical and psychological development period, full of passion and creativity. Research has shown that this period is a turbulent, contradictory, rough, and stormy period. Significant physical and psychological changes occur in adolescents. Adolescents may experience a range of psychological challenges and emotional issues (35). Adolescent MDD is caused by an interaction between genetic and environmental factors. For instance, sex hormones affect the brain by activating the development of the prefrontal cortex, amygdala, and hypothalamus, and hormone and induce physical changes, which may be associated with the increased risk of adolescent MDD (36, 37).

The level of Maresin-1 in adolescent MDD patients was significantly lower than in the HC group, implying that these patients may have insufficient inflammation resolution pathological features. Fish oil rich in ω-3 fatty acid has been reported to have the potential to alleviate depressive symptoms (38, 39), and the inflammation resolution substance Maresin-1 is the metabolite of ω-3 fatty acid *in vivo*. This suggests that Maresin-1 may have the potential as a novel diagnostic biomarker of adolescent MDD, which can help to further subdivide MDD patients with inflammatory characteristics for personalized and targeted treatment. After 6–8 weeks of fluoxetine treatment, the level of Maresin-1 in the peripheral blood of adolescent MDD patients significantly increased, with an improved HDRS-17 Score. Multivariate regression analysis revealed that a lower serum level of Maresin-1 was a risk factor for depressive symptom severity. Therefore, it is hypothesized that the circulatory status of Maresin-1 may be a predictor of adolescent MDD progression, which require further study.

IL-6, a pro-inflammatory cytokine, is primarily involved in the inflammation response and hematopoiesis process, as well as stimulating the proliferation of activated B cells, which are abundant in adipocytes. The synthesis and secretion of IL-6 can be influenced by inflammatory factors such as IFN-γ and TNF-α, with IL-6 potentially enhancing HPA axis activity (40, 41). The IL-6 level in the MDD group was significantly higher than in the HC group. Previous case–control and cross-sectional studies in the adolescent community reported an increased level of IL-6 in MDD patients (42, 43). Nevertheless, some studies did not identify this difference due to differences in the methods used. Some demonstrated the role of gender factors and the correlation between the high level of IL-6 and gender factors (44, 45). To avoid the heterogeneity of adolescent depressive symptoms, some studies focused on anhedonia and confirmed that the increased level of IL-6 was indeed related to anhedonia in adolescent MDD (46). Additionally, the level of IL-6 decreased significantly after treatment with Fluoxetine. This corroborates the previous studies, where IL-6 decreased from the baseline level after 4-week treatment with Fluoxetine. In that study, however, after 8 weeks of Fluoxetine treatment, the level of IL-6 reverted to the pre-treatment levels (47). Perić et al. (48) evaluated the effect of fluoxetine anti-depressant treatment and found that Chronic Social Isolation (CSIS) induced depression and anxiety-like behavior in rats and increased IL-6 protein levels in the central nervous system. On the other hand, fluoxetine treatment reversed depression and anxiety-like behavior and inhibited the activation of NF-κB and cytosolic IL-6 protein expression in CSIS rats. Fang et al. (49) Also, Fluoxetine might restrict the activation of reactive A1 astrocytes through the astrocyte 5-HT2BR/β-arrestin2 pathway and decrease IL-6 levels in the central nervous system.

This study showed that the circulating IL-1β in adolescent MDD patients was not significantly different from that of the HC group, consistent with previous studies (50, 51). However, depression severity and the IL-1β level were lower after a 6–8-week treatment by Fluoxetine. Regression analysis showed that IL-1β level was negatively correlated to depression severity. Similar to previous studies, IL-1β is primarily expressed in the CNS’s hypothalamus, cerebral cortex, and hippocampus and is produced mainly by neurons, microglia, and astrocytes in the brain (52). Moreover, IL-1β can induce inflammatory factors like IL-6 and TNF-α, with immune regulation and endocrine effects. Hence, it plays an extensive and essential role in the immune regulation of MDD. Based on clinical research and experimental findings, Pineda et al. (53) argued that cerebral and peripheral inflammation is a factor leading to MDD. However, they established the relationship between the polymorphism of IL1-β promoter and MDD, confirming a solid association between IL-1β and MDD. Goshen et al. (54) observed that IL-1β in the brain could mediate chronic stress-induced MDD, and the mice with the IL-1β type I receptor gene did not develop depressive-like behavior caused by chronic stress after the knockout. These results verified the essential functions of IL-1β in depression onset and progression. The peripheral levels of IL-1β were investigated in this study, but its concentration in the central nervous system could not be accessed. Nevertheless, animal studies have demonstrated that intraperitoneal LPS administration increased the inflammatory factor IL-1β in the hippocampus of mice and induced time-dependent microglia activation and depression-like behavior (55). In a study on sepsis mice with a cognitive impairment model, the importance of the NLRP3-IL-1β pathway in the transition from acute peripheral inflammation to chronic central neuritis was reported (56). The meta-analysis of Fluoxetine treatment efficacy in adult MDD patients also revealed that its effectiveness of Fluoxetine was associated with the levels of IL-6 and IL-1β in the peripheral blood (19).

The level of Maresin-1 in the peripheral blood of adolescent patients with MDD was associated with the levels of IL-6 and IL-1β in this study. Maresin-1 was negatively correlated with IL-1β and IL-6 while positively correlated with IL-4. Immature circulating monocyte level in MDD was higher than that in healthy controls (57). A recent study isolated the monocyte-derived macrophages *in vitro* (Mo-MФs) from the peripheral blood of adolescent patients with MDD and discovered that MDD patients had higher pro-inflammatory M1 polarization characteristics. Furthermore, the plasma from MDD patients had immunosuppressive effects on healthy donor macrophages, reducing the monocytes and B cells activation and T cell memory (58). Previous studies found that Maresin-1 enhanced alternative activation of CD11c − CD206+ (M2) macrophages while inhibiting polarization of CD11c + CD206− (M1) macrophages (59). This effect of Maresin-1 has a negative regulatory effect on inflammation response. Moreover, Maresin-1 has been confirmed to suppress the synthesis of pro-inflammatory cytokines in the anti-inflammatory effect. For instance, Maresin-1 inhibits the production of IL-1β (60) and TNFα (61). A study on human cells found that Maresin-1 inhibited the production of TNF-α and IL-6 by inhibiting the SIRT1/PGC-1α/PPARγ pathway (62). However, few studies have explored the mechanism of Maresin-1 on MDD human blood cells. Hence further investigations are needed.

In this study, the Maresin-1 level decreased, whereas IL-6 increased in adolescent MDD patients. Maresin-1 levels and IL-4 increased after 6–8 weeks of fluoxetine treatment in the acute phase, whereas IL-6 and IL-1β decreased. Despite the small number of enrolled participants, an imbalance between inflammation resolution and pro-inflammatory mediators was detected. Our findings demonstrated that the circulatory level of Maresin-1 might play a critical role in the development of depressive symptoms as well as drug treatment. Therefore, targeting Maresin-1 may aid in identifying novel diagnostic and therapeutic strategies that can be effectively applied to MDD patients and other inflammatory diseases, but *in vivo* and *in vitro* experiments further needed to confirm this relationship. Our findings suggest a disorder in the dissipation of inflammation in depressed adolescents, but our data need to be extended to cohort studies to confirm this hypothesis. Nevertheless, these findings could suggest that some adolescents have abnormal dissipation of inflammation and that these patients with reduced Maresin-1 responded well to fluoxetine treatment and benefited more.

## 5. Limitations of the study

This study has some limitations, including a small number of cases, use of a single treatment, and the low observation time points, which affect the evidence obtained for the mechanism of the observed changes. However, we controlled for the effects of factors such as sex and age, BMI, and other factors for depression and healthy controls. We still found that the adolescent family environment, parents’ perception of the condition, and compliance affected the treatment effect of adolescent depression. Therefore, even during the 6–8 week evaluation cycle, there were still many dropouts in the depression group. We only showed results for patients with good adherence. This study could not include the effects of factors such as family environmental factors and childhood adversity on reducing depressive symptoms and inflammation in adolescents, which will be refined in future research.

## Data availability statement

The original contributions presented in the study are included in the article/supplementary material, further inquiries can be directed to the corresponding authors.

## Ethics statement

The studies involving human participants were reviewed and approved by the ethics committee of the First Affiliated Hospital of Chongqing Medical University. Written informed consent to participate in this study was provided by the participants’ legal guardian/next of kin.

## Author contributions

TQ: analyzing the data and writing the paper. XL: helping modifying the report. JH and WC: participants enrollment screening. LS and CZ: processing and testing of blood samples. ZL, CT, and QY: assist with CRF data entry. LD and JG: conceived and designed the experiments, fund provider, writing—a review. All authors contributed to the article and approved the submitted version.

## Funding

This work was supported by Chongqing Science and Health Joint Medical Scientific Research Project (2020MSXM079).

## Conflict of interest

The authors declare that the research was conducted in the absence of any commercial or financial relationships that could be construed as a potential conflict of interest.

## Publisher’s note

All claims expressed in this article are solely those of the authors and do not necessarily represent those of their affiliated organizations, or those of the publisher, the editors and the reviewers. Any product that may be evaluated in this article, or claim that may be made by its manufacturer, is not guaranteed or endorsed by the publisher.
